# A novel hypoxia-related signature for predicting prognosis, immune characteristics, and therapeutic response in hepatocellular carcinoma

**DOI:** 10.1007/s12672-026-05691-w

**Published:** 2026-08-07

**Authors:** Chengting Wu, Yuanqin Du, Juhong Jia, Xinyuan Chen, Longda Wu, Yaran Zhao, Qian Peng, Jiazhi Li, Yanhua Lai, Dewen Mao, Yanfei Wei

**Affiliations:** 1 Guangxi University of Chinese Medicine, Nanning, China; 2 The First Affiliated Hospital of Guangxi University of Chinese Medicine, Nanning, China; 3 Guangxi Key Laboratory of Translational Medicine for Treating High-Incidence Infectious Diseases with Integrative Medicine, Nanning, China; 4 The People’s Hospital of Guangxi Zhuang Autonomous Region, Nanning, China

**Keywords:** Hepatocellular carcinoma, Hypoxia, Prognostic model, Immune microenvironment

## Abstract

**Objective:**

This study aimed to develop and validate a hypoxia-related gene signature for prognostic assessment in hepatocellular carcinoma (HCC) and to explore its associated biological characteristics and immune features. In addition, a self-established human tissue cohort was used to verify the expression stability of the identified signature genes.

**Methods:**

The TCGA-LIHC cohort was used as the training set to identify differentially expressed hypoxia-related genes associated with overall survival. Least absolute shrinkage and selection operator (LASSO) regression and Cox regression analyses were subsequently applied to construct a prognostic model. The predictive performance of the model was externally validated using the GSE14520 and GSE116174 cohorts. Functional enrichment and immune microenvironment analyses were performed to characterize the biological differences between risk groups. Furthermore, quantitative real-time PCR (qRT-PCR) was conducted in a human tissue cohort, including normal liver tissues, adjacent non-tumorous tissues, and HCC tissues, to validate the expression patterns of the four signature genes (TMEM45A, PPARGC1A, EFNA3, and STC2).

**Results:**

A four-gene hypoxia-related prognostic signature was established and successfully stratified patients into high- and low-risk groups. Patients in the high-risk group exhibited significantly poorer overall survival in both the training and validation cohorts. Functional enrichment analyses revealed that high-risk tumors were associated with activation of pathways related to cell-cycle progression, MYC targets, epithelial–mesenchymal transition, and metabolic reprogramming. Immune analyses demonstrated increased M0 macrophage infiltration and elevated expression of multiple immune checkpoint genes in the high-risk group. qRT-PCR validation further confirmed the differential expression patterns of the four signature genes in human HCC tissues.

**Conclusion:**

This four-gene hypoxia-related signature demonstrated robust prognostic performance across multiple independent cohorts and was associated with distinct metabolic and immune characteristics in HCC. qRT-PCR validation further supported the expression stability of the identified genes in human tissues. These findings provide a useful framework for prognostic stratification and future investigation of hypoxia-related biological mechanisms and therapeutic strategies in HCC.

**Supplementary Information:**

The online version contains supplementary material available at 10.1007/s12672-026-05691-w.

## Introduction

Hepatocellular carcinoma (HCC) is one of the most aggressive and lethal liver cancers worldwide and ranks as the fourth leading cause of cancer-related death [1]. Early detection can result in a 5-year survival rate exceeding 70%, and surgical resection generally yields favorable outcomes [2]. However, most patients are diagnosed at advanced stages, with a 5-year survival rate of only 12.5% in China [3]. The poor prognosis and high mortality of HCC are largely attributed to its subtle symptoms and indistinct clinical features, highlighting the urgent need to identify factors influencing patient outcomes to improve therapeutic benefit.

HCC frequently presents as multiple nodules, which may arise from intrahepatic metastasis or independent multicentric occurrences [4]. Although both normal liver and HCC tissues are highly vascularized, the rapid proliferation of tumor cells within nodules often leads to excessive oxygen consumption, resulting in a hypoxic tumor microenvironment. Indeed, HCC is among the most hypoxic solid tumors, with median oxygen levels in tumor tissues as low as 0.8% [5]. Accumulating evidence indicates that hypoxia-inducible factors (HIFs) contribute to HCC cell proliferation, angiogenesis, invasion, and metastasis [6, 7].

Based on these observations, we hypothesized that hypoxia holds prognostic significance in HCC. In this study, we systematically constructed a novel hypoxia-related prognostic signature and validated its utility as an independent predictor of HCC patient outcomes. Furthermore, we comprehensively evaluated the association of this hypoxia signature with immune cell infiltration, drug sensitivity, and potential immune checkpoints, and validated the expression of key genes in clinical samples. These findings provide a new basis for improving prognostic assessment and guiding therapeutic strategies in HCC.

## Materials and methods

RNA sequencing data and corresponding clinical details for HCC patients were retrieved from The Cancer Genome Atlas (TCGA) database (https://portal.gdc.cancer.gov/). The TCGA-LIHC dataset included 50 normal liver tissue samples and 369 HCC samples, which were utilized for differential expression analysis. After excluding samples with incomplete overall survival (OS) data, 363 HCC samples remained for subsequent investigation.

The GSE14520 dataset, sourced from the Gene Expression Omnibus (GEO) database, contained 241 normal tissues and 247 HCC samples for the purpose of differential expression analysis. After removing samples with missing OS data, 221 HCC samples were included for further examination. The GSE116174 dataset, which included 63 HCC samples with full OS data, was employed as an independent validation set.

**Fig. 1:**
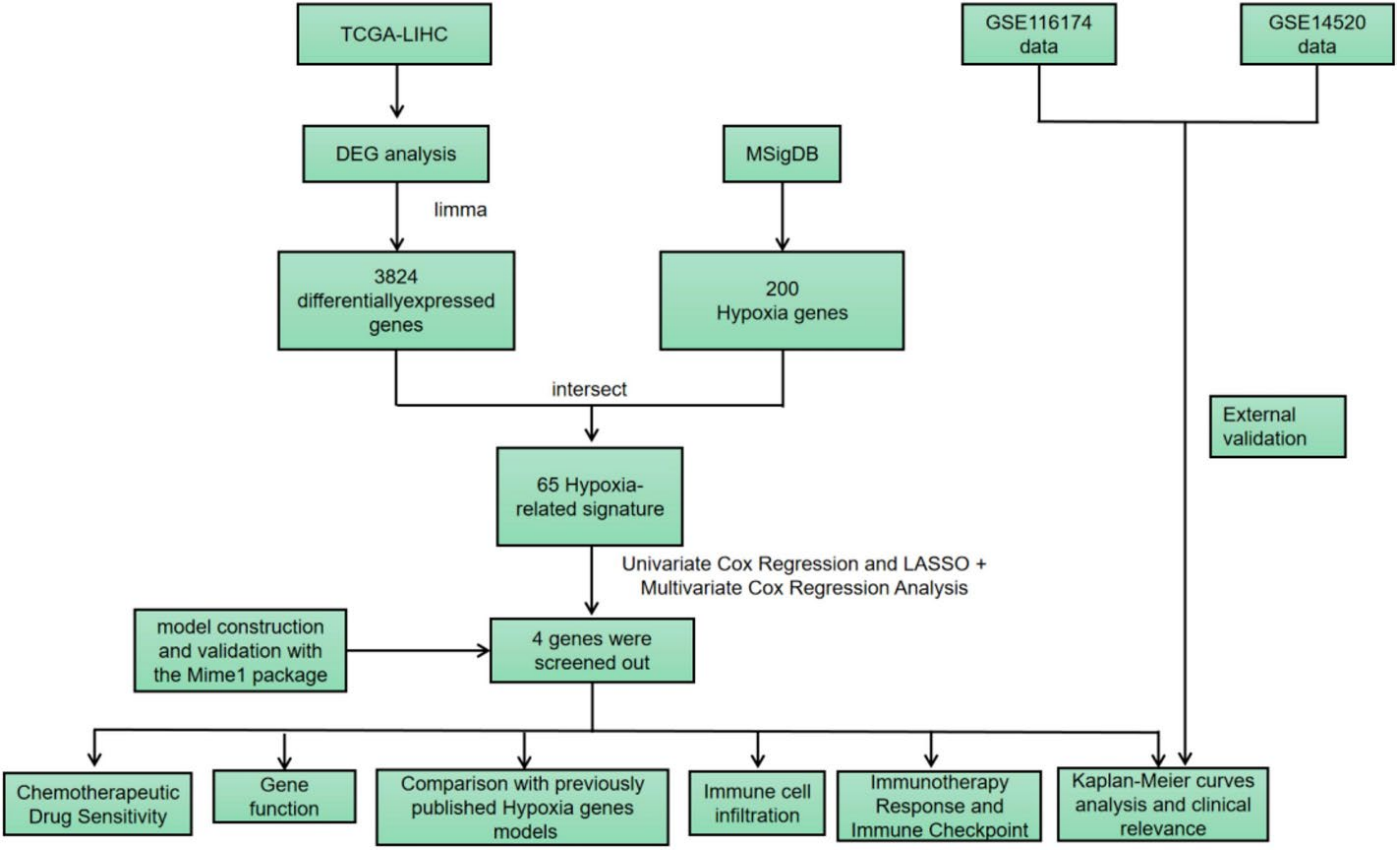
The workflow of this study, including the datasets and analytical methods used: *TCGA-LIHC* The Cancer Genome Atlas Liver Hepatocellular Carcinoma dataset, *GSE14520 and GSE116174* GEO datasets, *MSigDB* Molecular Signatures Database, *DEG* differentially expressed genes, and *LASSO* least absolute shrinkage and selection operator regression analysis

To identify hypoxia-associated genes in relation to HCC prognosis, 200 hypoxia-related genes were extracted from the Molecular Signatures Database (MSigDB) [8] (http://www.gsea-msigdb.org/gsea/msigdb). The overall study workflow is illustrated in Fig. 1.

### Construction and analysis of hypoxia-related genes (HRGs)

Differentially expressed genes (DEGs) between tumor and normal samples in the TCGA dataset were initially identified using the R package “limma,” with cutoffs of |log2(FC)|> 1 and a false discovery rate (FDR) < 0.05. These thresholds were selected to balance statistical significance and biological relevance and have been widely adopted in transcriptomic biomarker discovery studies. The DEGs were then cross-referenced with known hypoxia-related genes (HRGs) to identify hypoxia-associated DEGs in HCC. Univariate Cox regression analysis was performed on these intersected genes in the TCGA cohort to identify genes significantly linked to overall survival (OS, P < 0.05). To reduce the number of features and prevent overfitting, LASSO regression was applied for further refinement of candidate genes. The genes selected by LASSO were then incorporated into a multivariate Cox regression model to generate a hypoxia-related prognostic risk score.

To determine the best predictive model, the R package “Mime1” [9], which integrates ten common machine learning methods, was utilized. Model performance was assessed by comparing concordance indices (C-index) and time-dependent area under the curve (AUC) values at 1-, 3-, and 5-year intervals in both the training and validation cohorts. The model with the most consistent performance across datasets was selected for further analysis.

Patients were classified into high- and low-risk groups based on the median risk score using the “survminer” and “survival” packages. Kaplan–Meier survival analysis and time-dependent ROC curves with 1-, 3-, and 5-year AUC values were employed to evaluate model accuracy. Univariate Cox regression analyses were conducted in the TCGA, GSE14520, and GSE116174 datasets to assess the prognostic significance of each factor. Hazard ratios (HRs), 95% confidence intervals (CIs), and P values were computed, and a meta-analysis using fixed- and random-effects models was performed to examine the consistency and robustness of the features across datasets. Statistical analyses were conducted in R, with significance defined as *P* < 0.05.

Lastly, samples from TCGA, GSE14520, and GSE116174 were grouped into high- and low-risk categories based on median risk scores. Risk score distributions, survival statuses, and gene expression patterns were analyzed. Principal component analysis (PCA) was used to examine differences in multidimensional distributions between the groups. To further validate the model's performance, it was compared with previously published HRG models based on HR, C-index, and AUC metrics.For comparative analyses, previously published hypoxia-related prognostic signatures were reconstructed according to their reported gene compositions and regression coefficients. Risk scores for each published signature were recalculated in the TCGA, GSE14520, and GSE116174 cohorts using the same normalized expression matrices. Subsequently, HRs, C-index values, and time-dependent AUCs were evaluated using identical analytical procedures and performance metrics, enabling a standardized comparison of prognostic performance across different models.

### Construction and validation of the nomogram

To assess whether the risk score model could independently predict prognosis in HCC, both univariate and multivariate Cox regression analyses were conducted on clinical features and risk groups in the TCGA, GSE14520, and GSE116174 datasets using the “survival” R package. A nomogram was then developed using the “rms” R package to predict the 1-, 2-, and 3-year overall survival (OS) for HCC patients.

### Functional enrichment analysis

Based on the median HRG risk score, samples were categorized into high- and low-risk groups. Differentially expressed genes (DEGs) between these two groups were identified using the “limma” R package to investigate differences in biological functions and molecular pathways. Gene Ontology (GO) and Kyoto Encyclopedia of Genes and Genomes (KEGG) enrichment analyses were performed on the DEGs using the “clusterProfiler” R package. Additionally, gene set enrichment analysis (GSEA) was carried out with reference gene sets from the Molecular Signatures Database (MSigDB, symbols.gmt v7.0) using the “clusterProfiler” and “enrichplot” packages to uncover potential biological pathways and functional processes.

### Protein–protein interaction (PPI) network construction

The differentially expressed genes identified through risk score stratification were uploaded to the STRING database (https://cn.string-db.org/) with a confidence score cutoff of 0.9 to build the protein–protein interaction (PPI) network. The network was then visualized using Cytoscape to depict the interactions between the selected genes.

### Immune infiltration and tumor microenvironment analysis

To evaluate immune cell infiltration and tumor microenvironment (TME) composition in different risk groups, we conducted immune infiltration analysis using multiple algorithms via the “IOBR” R package. Raw TPM expression matrices were log2-transformed, and genes with low expression (average expression < 0.5) were excluded. Eight widely used algorithms—MCPcounter, EPIC, xCell, CIBERSORT, IPS, quanTIseq, ESTIMATE, and TIMER—were applied to assess immune cell infiltration and TME components for each sample.

Additionally, single-sample gene set enrichment analysis (ssGSEA) was performed using immune-related gene signatures from IOBR to evaluate immune functional status and pathway activity in each sample. The results from all algorithms were normalized and combined into a unified immune infiltration feature matrix. Based on the median risk score, samples were divided into high- and low-risk groups, and differences in immune cell subpopulations and immune pathway activity between the groups were compared. Heatmaps were generated to visualize immune infiltration feature patterns across risk groups.

Furthermore, differential expression of common immune checkpoint genes between subgroups was analyzed using the “limma” package. To predict the response to immunotherapy, TIDE scores were calculated for each sample using the TIDE algorithm, and the scores were compared between high- and low-risk groups. The TIDE algorithm estimates the potential response to immune checkpoint inhibitors, with lower scores suggesting a better predicted efficacy of immunotherapy. TIDE data were obtained from the online database (http://tide.dfci.harvard.edu/faq/).

### Drug sensitivity analysis

Drug sensitivity in high- and low-risk HCC patients based on HRGs was evaluated using the “oncoPredict” R package. This tool predicts drug response from gene expression data, serving as a guide for personalized treatment. A total of 198 drugs were analyzed, and the half-maximal inhibitory concentration (IC50) for each patient was computed. IC50 indicates the drug concentration needed to inhibit 50% of cell growth, reflecting the drug's effectiveness on cells. By comparing IC50 values between high- and low-risk groups, potential therapeutic agents suitable for each risk group were identified.

### RNA extraction, reverse transcription, and quantitative real-time PCR (qRT-PCR)

For gene expression analysis, liver tissue samples were homogenized using the KZ-III-F low-temperature grinding system (Servicebio, China). Total RNA was extracted from the tissue homogenates with TRIzol reagent according to standard protocols. The isolated RNA was then reverse transcribed into cDNA using the HyperScript™ III Reverse Transcription Kit. Quantitative real-time PCR (qRT-PCR) was conducted on a LightCycler 480II system (Roche, Switzerland) with 2 × S6 Universal SYBR qPCR Mix. Primers were designed based on gene sequences from GenBank, and the specific sequences are listed in Table S1.

### Patients and specimens

This study included 12 pairs of HCC tissues and matched adjacent non-tumor tissues, which were collected from patients who underwent liver transplantation at Guangxi Zhuang Autonomous Region People’s Hospital between 2013 and 2024. All tissue samples were stored following the institutional guidelines at the Guangxi Zhuang Autonomous Region People’s Hospital Biobank (Biobank approval number: Guoke Yiban Shenzi [2023] BC0039). The study was approved by the Ethics Committee of Guangxi University of Traditional Chinese Medicine, and written informed consent was obtained from all patients for the use of their tissues.

### Statistical analysis

All statistical analyses were conducted using R software (version 4.4.2) and the MicrobeX online platform. Various R packages, including limma, survival, and caret, were utilized. Unless otherwise specified, all statistical tests were two-sided. Statistical significance was indicated as follows: *P < 0.05, **P < 0.01, and ***P < 0.001. A *p* value < 0.05 was considered statistically significant.

## Results

### Construction and validation of HRGs

**Fig. 2:**
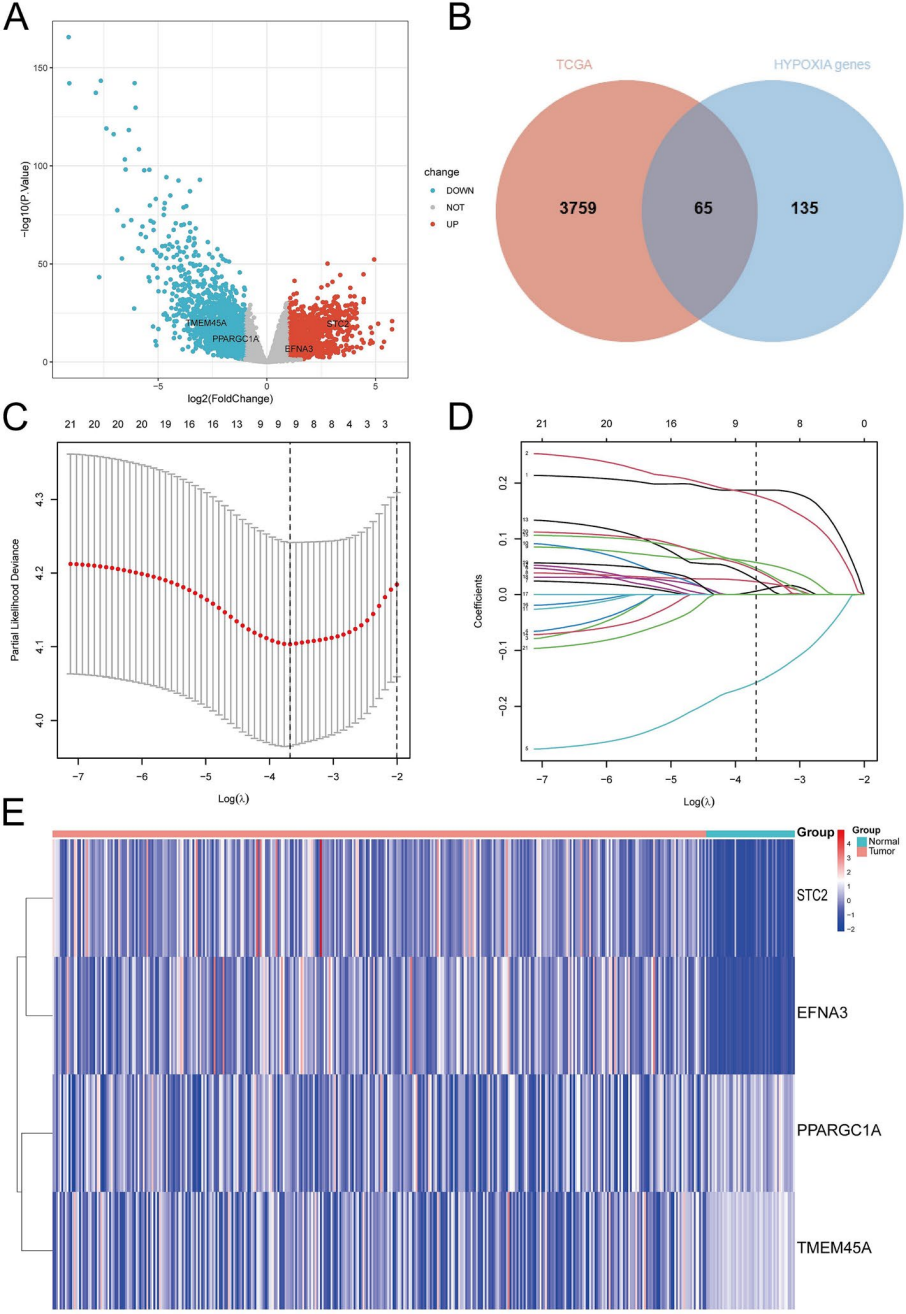
Differential gene expression analysis results. **A** Volcano plot showing differentially expressed genes between normal and tumor tissues in the TCGA dataset; **B** Venn diagram of differentially expressed genes and hypoxia-related genes (HRGs); **C** LASSO regression coefficient profiles of 21 HRGs; **D** Parameter tuning plot for the LASSO model; **E** Heatmap showing the expression of four feature genes in normal and tumor tissues in the TCGA dataset

A total of 419 samples, including 50 normal liver tissues and 369 HCC samples, were obtained from the TCGA database. Differential expression analysis revealed 3,824 differentially expressed genes (DEGs) between tumor and normal tissues (Fig. 2A). Next, 200 hypoxia-related genes (HRGs) were obtained from the MSigDB database and intersected with the DEGs, resulting in 65 differentially expressed HRGs (Fig. 2B; Supplementary Table S1).

Univariate Cox regression analysis of these 65 HRGs identified 21 genes significantly associated with overall survival in the TCGA cohort (Supplementary Table S2). To further reduce overfitting and select the most informative prognostic features, LASSO Cox regression analysis was performed. Using the optimal penalty parameter determined by ten-fold cross-validation, nine candidate genes (STC2, EFNA3, PPARGC1A, ADM, HMOX1, TMEM45A, MAFF, SLC2A5, and STC1) were retained for subsequent analysis (Supplementary Figure S3–S4 and Supplementary Table S3).

Subsequently, an AIC-based stepwise multivariate Cox regression analysis was conducted to optimize model performance and eliminate redundant variables. Five genes (ADM, HMOX1, MAFF, SLC2A5, and STC1) were sequentially excluded during the stepwise selection process because they did not improve the overall model fit. Ultimately, four hypoxia-related genes (TMEM45A, PPARGC1A, EFNA3, and STC2) were retained to construct the final prognostic signature (Supplementary Table S4). Heatmaps illustrating the expression patterns of these four genes in TCGA samples are shown in Fig. 2C–E.

**Fig. 3:**
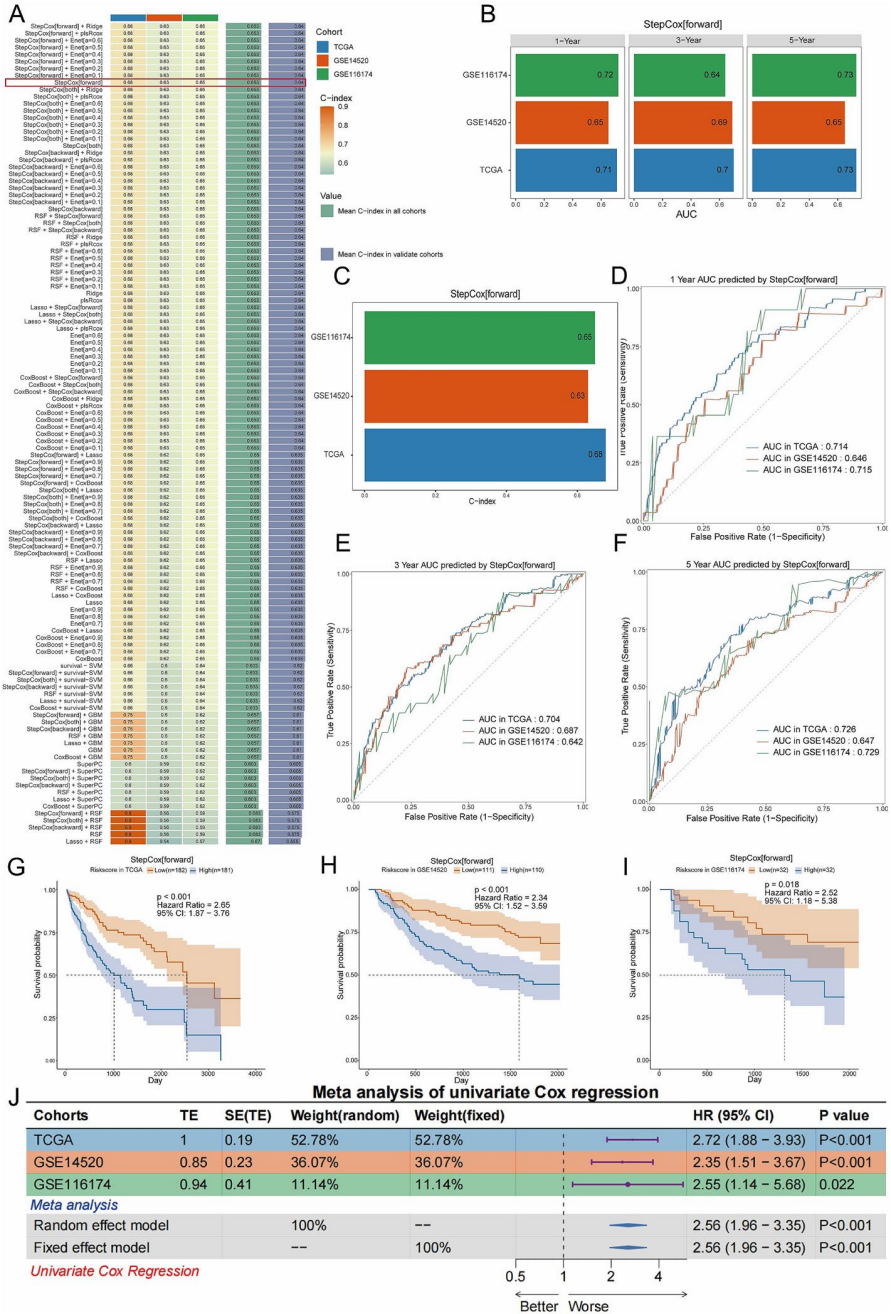
Model construction and validation. **A** Predictive models constructed using multiple machine learning algorithms; **B** AUC values of the StepCox[forward] model for predicting 1-, 3-, and 5-year survival outcomes across different datasets; **C** C-index values of the StepCox[forward] model in various datasets; **D**–**F** ROC curves for 1-, 3-, and 5-year survival predictions using the StepCox[forward] model in each dataset; **G**–**I** Kaplan–Meier survival curves showing overall survival (OS) differences between high- and low-risk groups in the TCGA, GSE14520, and GSE116174 cohorts. Dashed lines indicate median OS. High: high-risk group based on hypoxia-related gene signature (nHRGs); Low: low-risk group based on nHRGs; **J** Meta-analysis results of univariate Cox regression

Using the “Mime1” machine learning R package, a total of 117 predictive models were trained on the TCGA dataset, and the concordance index (C-index) for each model was computed in both the training and validation cohorts (Fig. 3A). The StepCox[forward] model achieved the best performance with a relatively simple structure, demonstrating average C-index values of 0.653 and 0.640 in the training and validation sets, respectively (Fig. 3A). Further evaluation in the TCGA training set and two independent validation cohorts, GSE14520 and GSE116174, yielded C-index values of 0.68, 0.63, and 0.65, respectively (Fig. 3C). Additionally, the area under the curve (AUC) values for predicting 1-, 3-, and 5-year overall survival (OS) were calculated for all models (Figure S1). The StepCox[forward] model showed consistently high AUC values across all time points and demonstrated strong stability and generalizability across multiple datasets (Fig. 3B).

Thus, the StepCox[forward] model was selected as the optimal model, and its AUC values were presented (Fig. 3C). To assess its prognostic performance, ROC curves for 1-, 3-, and 5-year OS were plotted in both the training and validation cohorts (Figs. 3D–F). Patients were classified into high- and low-risk groups based on the median risk score calculated from the StepCox[forward] model in the Mime1 package. The risk score formula was defined as follows:

Risk Score =  − 0.2015245 × PPARGC1A expression + 0.2617897 × STC2 expression + 0.2140888 × EFNA3 expression + 0.1018088 × TMEM45A expression.

Kaplan–Meier survival analysis showed that high-risk patients in the training cohort had significantly worse OS (Fig. 3G). Log-rank tests confirmed significant survival differences between the high- and low-risk groups, with similar results observed in the validation cohorts (Figs. 3H–I).

To further assess the prognostic value of the selected features across datasets, univariate Cox regression analyses were performed in each cohort, followed by a meta-analysis to integrate the results. Univariate Cox regression calculated the hazard ratio (HR) for each feature with respect to OS. In the training cohort, the feature had an HR of 2.72 (95% CI 1.88–3.91, *P* < 0.001), indicating a significant relationship with OS (Fig. 3J). This feature remained statistically significant in all validation cohorts, with *P* values < 0.05.

For the meta-analysis, both random-effects and fixed-effects models were used to combine results across different datasets. The random-effects model addressed potential heterogeneity, while the fixed-effects model assumed no such variability. The random-effects model produced a hazard ratio (HR) of 2.56 (95% CI 1.96–3.35, *P* < 0.001), showing that this feature consistently and significantly impacted survival prognosis across multiple datasets. This meta-analysis further confirmed the robustness and strong predictive power of this feature across different cohorts.

### Risk model evaluation

**Fig. 4:**
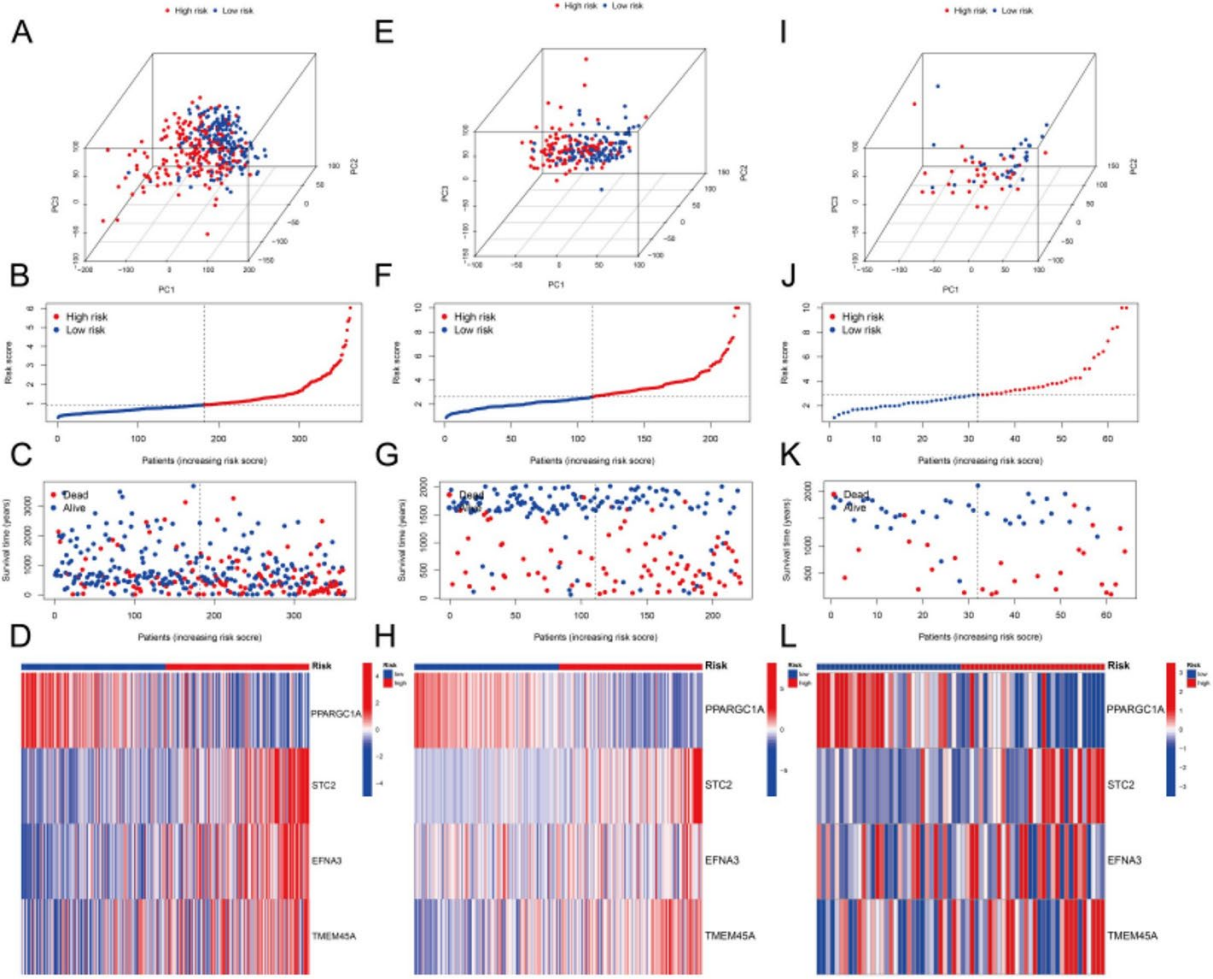
Performance of the prognostic model in high- and low-risk subgroups across the TCGA, GSE14520, and GSE116174 cohorts. **A**–**D** PCA three-dimensional analysis, risk score distribution, relationship between survival time and survival status, and heatmap of model gene expression in risk subgroups in the TCGA cohort. **E**–**H** PCA three-dimensional analysis, risk score distribution, relationship between survival time and survival status, and heatmap of model gene expression in risk subgroups in the GSE14520 cohort. **J**–**L** PCA three-dimensional analysis, risk score distribution, relationship between survival time and survival status, and heatmap of model gene expression in risk subgroups in the GSE116174 cohort

Patients in the TCGA, GSE14520, and GSE116174 cohorts were divided into high- and low-risk groups based on the median risk score. Principal component analysis (PCA) revealed clear separation between high- and low-risk patients in all three cohorts (Fig. 4A, E, I). Further analysis of gene expression levels, survival time, survival status, and risk score distributions was conducted. In the TCGA cohort (Fig. 4B–D), high-risk patients showed higher expression levels of the five signature genes and increased mortality rates. Similar trends were observed in the GSE14520 (Fig. 4F–H) and GSE116174 (Fig. 4J–L) cohorts.

Overall, these findings suggest that the prognostic model performs accurately and reliably across multiple independent datasets.

### Comparison with published hypoxia-related prognostic models

A review of recent literature identified eight hypoxia-related gene prognostic models. For each model, the associated genes, regression coefficients (Coef), and PubMed ID (PMID) were compiled (Tables 1 and S2). This summary serves as a reference to assess the performance of our model and allows for a thorough comparison with previously published models.

**Table 1:** Comparison of the hypoxia-related gene prognostic model constructed in this study with published models

Model	Gene list	References (PMID)
Our model	PPARGC1A,STC2,EFNA3,TMEM45A	–
Signature1	HMOX1,TKTL1,TPI1,ENO2,LDHA,SLC2A1	35116696 [10]
Signature2	GLP2R, C7, IL1RL1, CXCL6, CHST4, CLEC1B, GPR182, INMT	36307751 [11]
Signature3	ANLN, CBX2, DLGAP5, FBLN2, FTCD, HMOX1, IGLV1-44, IL33, LCAT, LPCAT1, MKI67, PFN2, RNF145, S100A9, SPP1	37481543 [12]
Signature4	HILPDA, EPO, SLC2A1, PSMA7, CREB1	39219818 [13]
Signature5	FDPS, SRM, NDRG1	37406019 [14]
Signature6	LDHA, KDELR3, CDKN1C, SLC2A1, NDRG1, VHL, SCARB1	33912215 [15]
Signature7	PDSS1,SLC7A11,CDCA8	32887635 [16]
Signature8	DCN,DDIT4,NDRG1,PRKCA	34093992 [17]

**Fig. 5:**
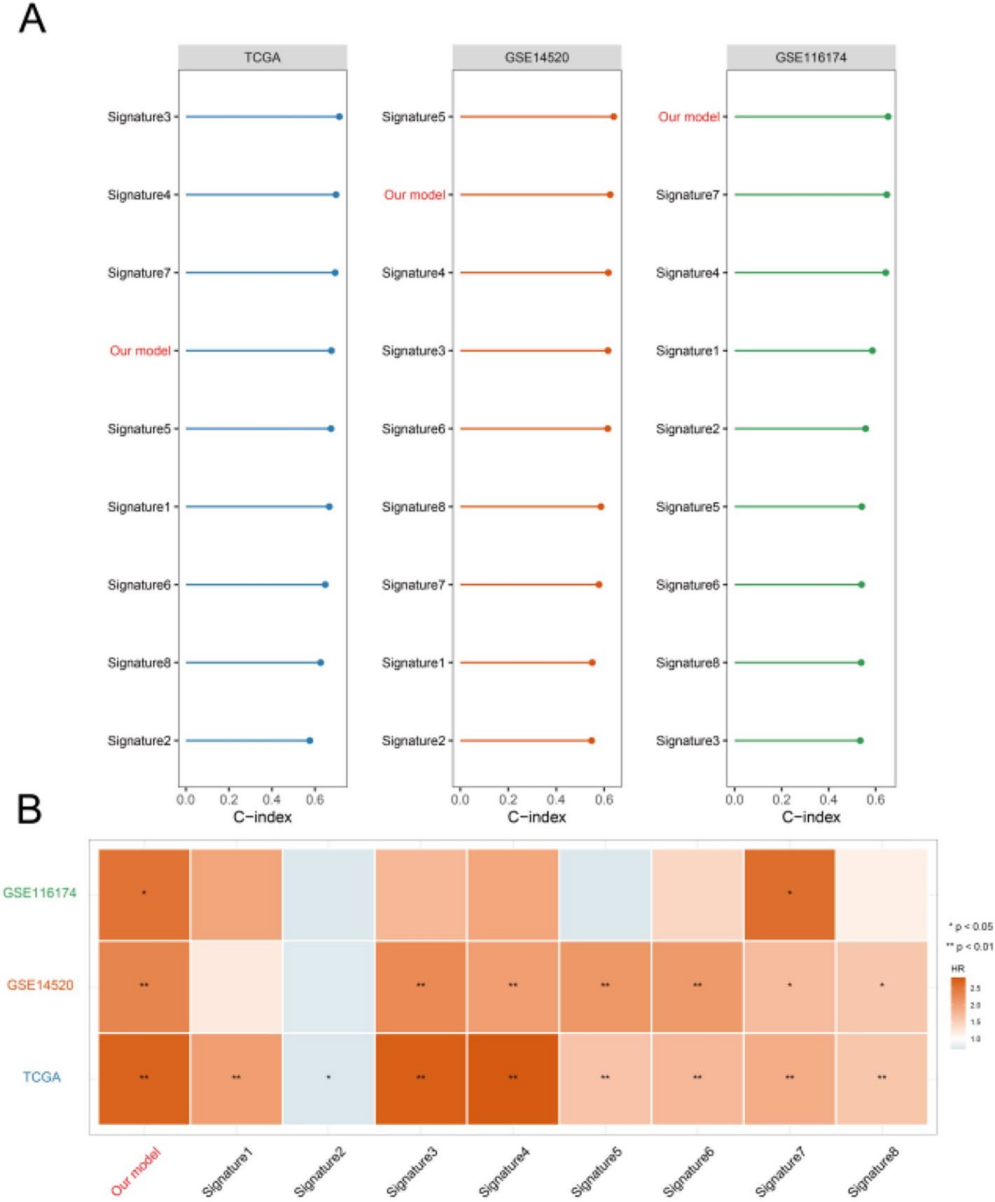
Comparison with published hypoxia-related gene models. **A** C-index comparison of each model across different datasets. **B** Heatmap of hazard ratios (HRs) for each model across datasets, with darker colors indicating higher HR values. Statistical significance is indicated by symbols: *P < 0.05, **P < 0.01

The results showed that, compared to existing prognostic models, our constructed model outperformed others in both the average C-index and the C-index of the validation cohorts (Fig. 5A). Similar patterns were observed for the 1-, 3-, and 5-year survival AUC values (Fig. S2). To further assess the prognostic ability of our model, it was compared with eight published hypoxia-related gene (HRG) prognostic models for HCC (Signatures 1–8, Table 1). In the heatmap (Fig. 5B), hazard ratios (HRs) are represented by different colors, with darker shades indicating higher HRs. Statistical significance is marked with symbols: **P < 0.01, *P < 0.05.

In comparative analyses across the training set and three validation cohorts, our model demonstrated robust prognostic predictive performance. In the TCGA cohort, the hazard ratio (HR) was close to 2.5, with high statistical significance (*P* < 0.01), clearly distinguishing between high- and low-risk patients. Similar consistent and significant results were seen in the two external validation cohorts, GSE14520 and GSE116174.

In contrast, while the eight previously published models showed statistical significance in the TCGA training set, their performance in the validation cohorts was generally less impressive. In the GSE116174 cohort, only Signature 7 maintained statistical significance, while the other models did not.

Overall, the comparative analyses across the three datasets highlighted that our model exhibited more stable and reliable predictive performance, as reflected in its C-index, AUC, and HR values, further supporting its robustness and superiority for prognostic prediction in HCC.

### Construction and validation of the nomogram

**Fig. 6:**
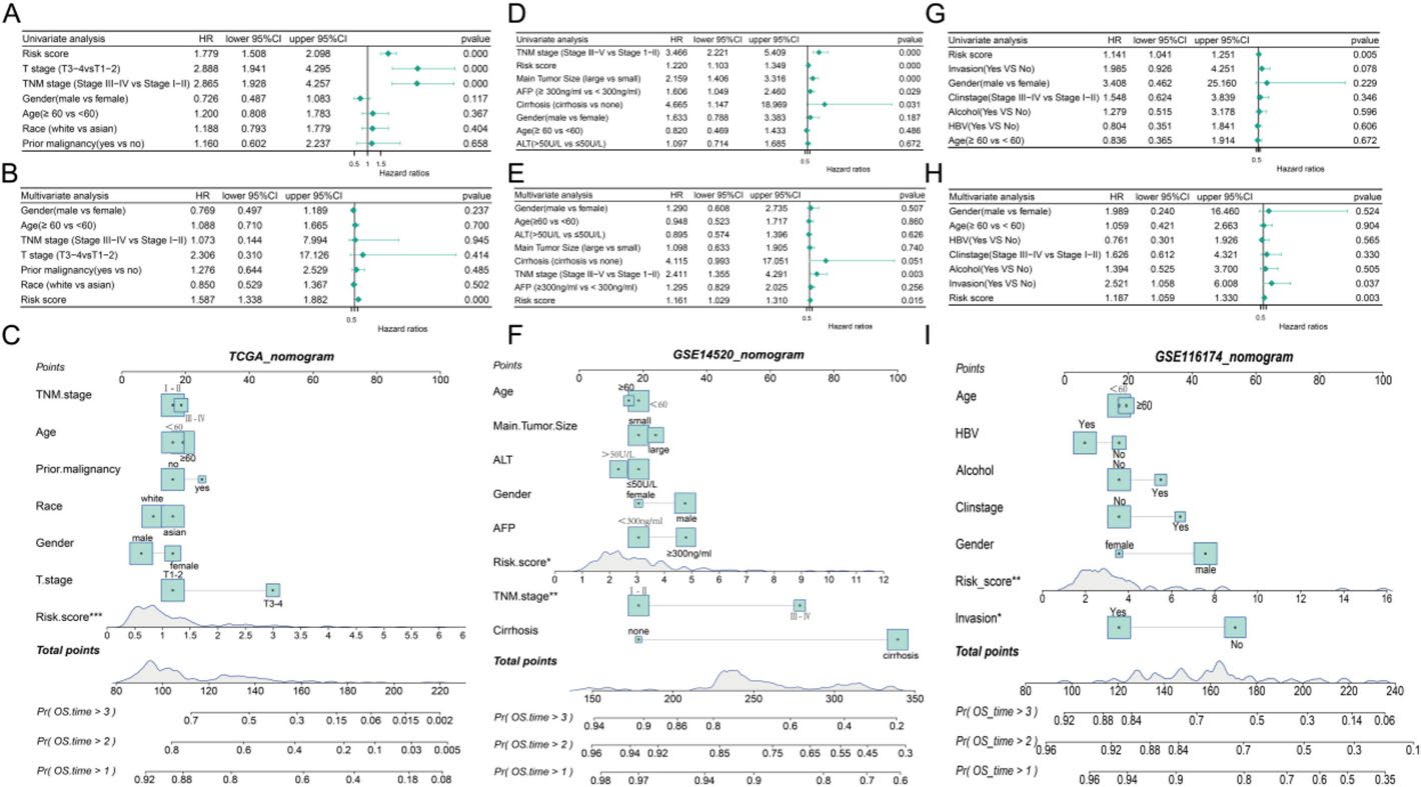
Construction and validation of the nomogram. **A**–**C** Forest plots of univariate and multivariate Cox regression analyses for risk score and clinical features, and the nomogram based on the risk score in the TCGA cohort. **D**–**F** Forest plots of univariate and multivariate Cox regression analyses for risk score and clinical features, and the nomogram based on the risk score in the GSE14520 cohort. **G**–**I** Forest plots of univariate and multivariate Cox regression analyses for risk score and clinical features, and the nomogram based on the risk score in the GSE116174 cohort

To evaluate whether the risk score and other clinical features could function as independent prognostic factors for HCC, univariate and multivariate Cox regression analyses were conducted. In the TCGA clinical dataset, univariate analysis revealed that the risk score, T stage, and TNM stage were significantly associated with patient prognosis (*P* < 0.05) (Fig. 6A). In multivariate analysis, only the risk score remained statistically significant (Fig. 6B), suggesting that it may serve as an independent prognostic factor for HCC.

A nomogram integrating the risk score and clinical features was subsequently developed based on the TCGA cohort (Fig. 6C). The nomogram demonstrated satisfactory predictive performance and calibration for predicting 3-year overall survival in patients with HCC.

Subsequent analyses were conducted using clinical data from the external validation cohorts, GSE14520 and GSE116174. In the GSE14520 cohort, univariate Cox analysis showed that TNM stage, alpha-fetoprotein (AFP) level, tumor size, presence of cirrhosis, and risk score were significantly associated with prognosis (*P* < 0.05) (Fig. 6D). Multivariate analysis confirmed that both the risk score and TNM stage independently predicted prognosis (Fig. 6E). In the GSE116174 cohort, univariate analysis identified the risk score as the only factor significantly associated with prognosis (*P* < 0.05) (Fig. 6G), whereas multivariate analysis identified tumor invasion status and risk score as independent prognostic factors (Fig. 6H).

Nomograms integrating the risk score and clinical features were also constructed for the two external validation cohorts and compared with the TCGA-based nomogram (Figs. 6F, I). These findings further support the robustness and predictive value of the nomogram across multiple independent cohorts. Although the nomogram demonstrated favorable predictive performance, decision-curve analysis and clinically actionable risk thresholds were not evaluated in the current study and therefore require further investigation in future prospective studies.

Importantly, subgroup analyses stratified by tumor stage demonstrated that the risk score could further distinguish high- and low-risk patients within the same stage, suggesting that it captures prognostic information beyond conventional clinical staging. These results support that the risk score reflects hypoxia-related biology rather than merely advanced-stage tumor characteristics.

### Functional enrichment analysis of differentially expressed genes (DEGs) between high- and low-risk groups

To explore the potential biological functions of differentially expressed genes (DEGs) between high- and low-risk groups in the hypoxia-related HCC prognostic model, Gene Ontology (GO) functional enrichment and Kyoto Encyclopedia of Genes and Genomes (KEGG) pathway analyses were conducted. Additionally, gene set enrichment analysis (GSEA) was performed to uncover the mechanisms underlying HCC development and progression.

**Fig. 7:**
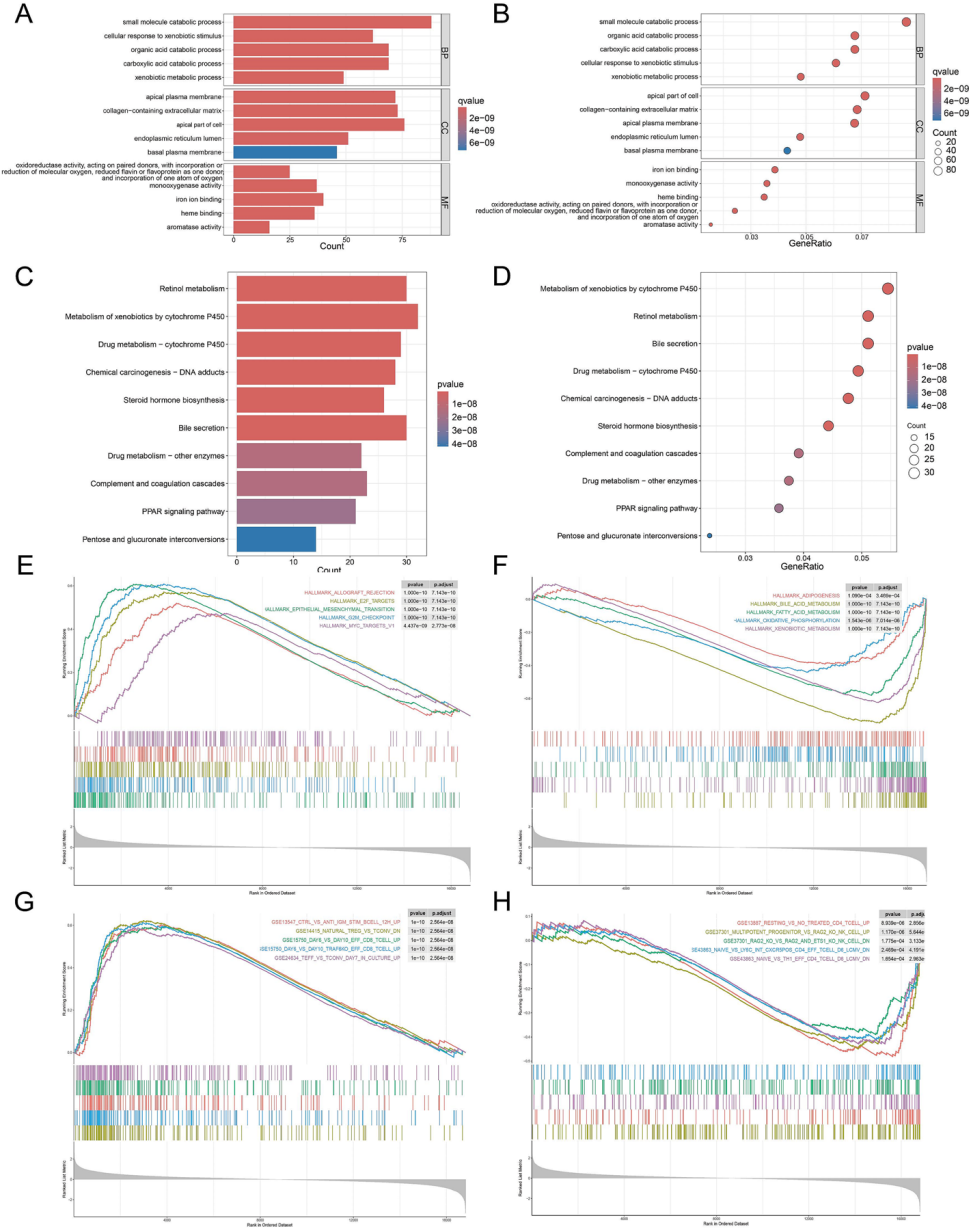
Functional enrichment analysis of differentially expressed genes (DEGs) between risk score subgroups. **A**, **B** GO enrichment analysis of DEGs between high- and low-risk groups. **C**, **D** KEGG pathway enrichment analysis of DEGs between high- and low-risk groups. **E**, **F** GSEA results for the Hallmark gene set. **G**, **H** GSEA results for the C7 immune-related gene set

GO enrichment analysis revealed that the DEGs were mainly involved in biological processes related to metabolism and cellular stress, such as the “small molecule catabolic process,” “organic acid catabolic process,” “xenobiotic metabolic process,” and “cellular response to xenobiotic stimulus.” Cellular component enrichment showed significant association with structures like the “apical plasma membrane,” “endoplasmic reticulum lumen,” and “basement membrane,” indicating potential roles in material transport and stress regulation (Fig. 7A, B).

KEGG pathway analysis further highlighted that these genes were mainly involved in pathways such as “cytochrome P450-mediated xenobiotic metabolism,” “retinol metabolism,” “steroid hormone biosynthesis,” “bile secretion,” and the “PPAR signaling pathway,” all of which are critical for tumor metabolic regulation. These findings suggest that under hypoxic conditions, HCC may gain a survival advantage through metabolic reprogramming (Fig. 7C, D).

GSEA revealed significant differences between high- and low-risk groups in terms of metabolic reprogramming and immune responses related to the tumor. In the high-risk group, enriched pathways included allograft rejection, indicating immune overactivation; E2F targets and the G2/M checkpoint, suggesting active tumor cell proliferation; epithelial-mesenchymal transition (EMT), implying increased invasive and metastatic potential; and MYC targets, reflecting activation of the MYC signaling pathway that regulates cell growth and metabolism (Fig. 7E). Immune analysis using the MSigDB C7 gene set revealed that the high-risk group was enriched in immune pathways related to B cell activation, CD8⁺ T cell activation, and regulatory T cell dysfunction, suggesting an immune-activated or inflammatory microenvironment that may contribute to immune evasion and tumor progression (Fig. 7G).

In contrast, GSEA for the low-risk group showed significant enrichment in pathways related to metabolic function and mitochondrial activity, such as fatty acid biosynthesis, bile acid metabolism, fatty acid metabolism, oxidative phosphorylation, and xenobiotic metabolism. This suggests that low-risk patients may have a more stable metabolic state and better mitochondrial function, which may help them adapt more effectively to hypoxic conditions (Fig. 7F). Immune-related pathways from the MSigDB C7 gene set showed that the low-risk group was primarily enriched in pathways associated with CD4⁺ T cell resting and immune homeostasis, indicating a relatively suppressed immune microenvironment that could mitigate the impact of excessive inflammation on tumor progression (Fig. 7H).

In summary, the functional enrichment analyses suggest that the DEGs between high- and low-risk groups are involved in key biological processes and pathways, including metabolic regulation, cell cycle progression, EMT, and modulation of the immune microenvironment. These mechanisms may contribute to HCC progression by influencing tumor cell proliferation, invasion, immune evasion, and are closely associated with hypoxic conditions.

### Construction of the PPI network

**Fig. 8:**
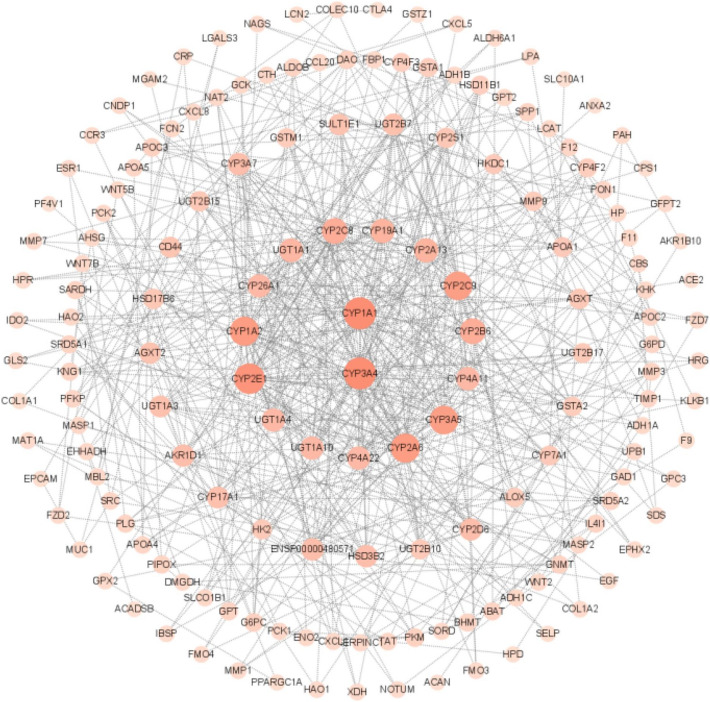
Protein–protein interaction (PPI) network of risk-associated differentially expressed genes

Differentially expressed genes (DEGs) between high- and low-risk groups were first identified. Subsequently, a protein–protein interaction (PPI) network of these DEGs was constructed using the STRING database and visualized with Cytoscape software (Fig. 8A).

### Correlation analysis between risk score and tumor immune microenvironment

**Fig. 9:**
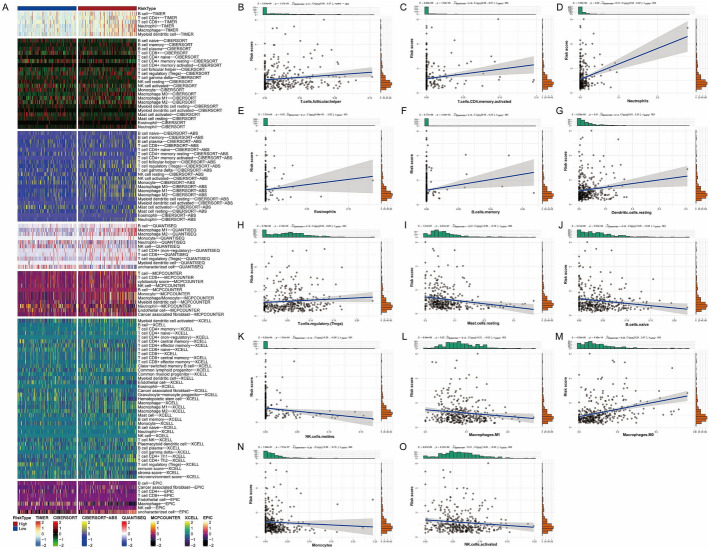
Correlation analysis between risk score and immune cell infiltration as well as tumor immune microenvironment features. **A** Heatmap showing infiltration levels of various immune cells across risk score subgroups in the tumor microenvironment. **B**–**O** Correlation plots between risk score and the abundance of different immune cell types

In the immune infiltration and tumor microenvironment heatmaps, the high-risk group generally showed higher infiltration levels of various immune cell types, with macrophages being notably upregulated. To investigate the relationship between immune cell infiltration and risk score, Spearman’s rank correlation coefficient (ρ) was calculated to assess the non-linear associations between the risk score and the infiltration levels of 22 immune cell types. The analysis revealed a significant negative correlation between the risk score and monocyte infiltration (ρ = − 0.26, P = 7.51e-07) and resting mast cells (ρ = − 0.27, P = 2.91e-07) (Fig. 9C, F). In contrast, a significant positive correlation was found between M0 macrophage infiltration and the risk score (ρ = 0.38, P = 4.95e-14) (Fig. 9H). Correlations with other immune cell types were relatively weak (Fig. 9).

### Analysis of risk score in relation to immune function, immune checkpoints, and drug sensitivity

**Fig. 10:**
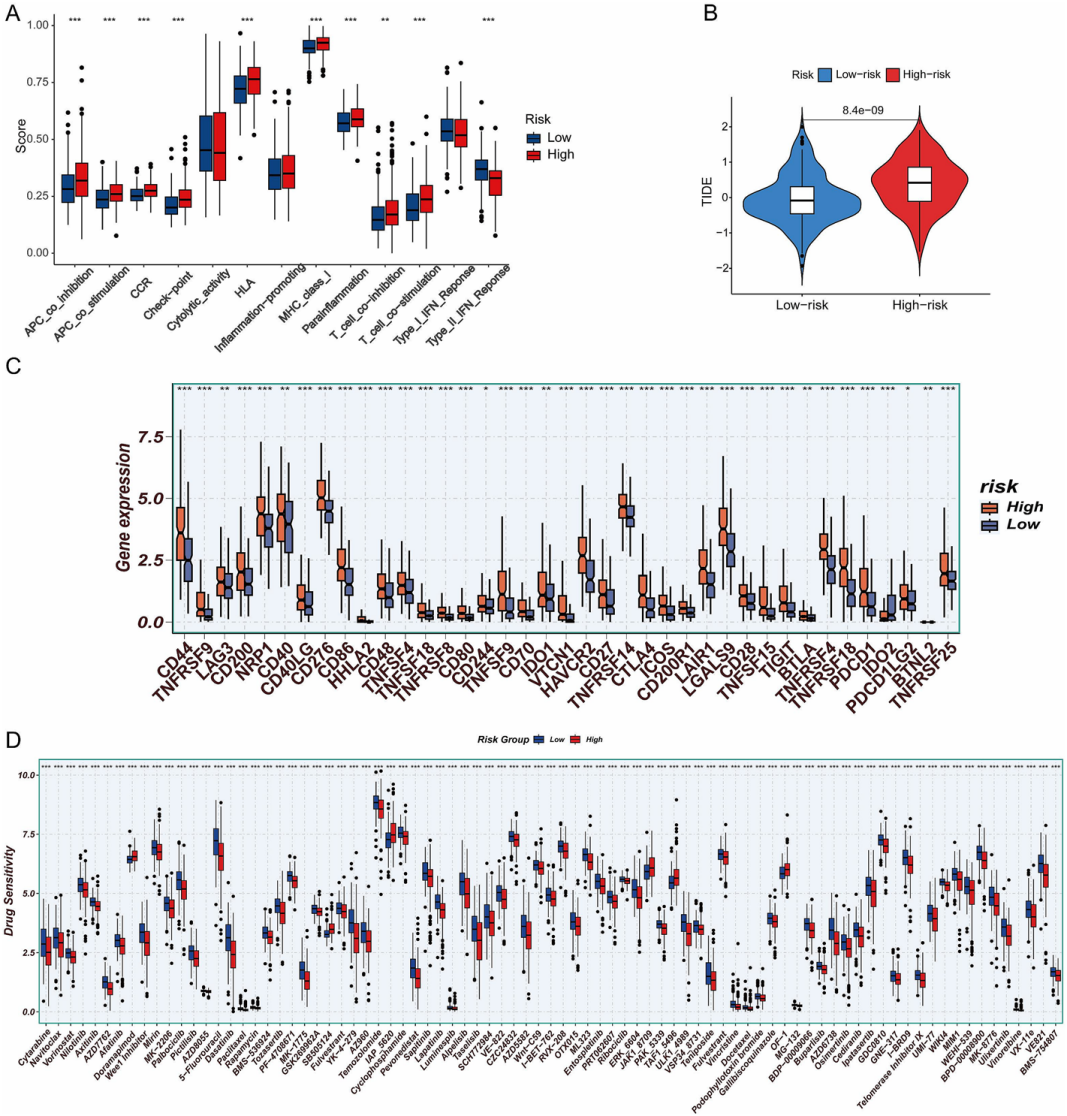
Association analysis of risk score with immune function, immune evasion, immune checkpoint expression, and drug sensitivity. **A** Comparison of immune function scores between different risk groups. **B** Differences in TIDE scores between high- and low-risk groups, evaluating their immune evasion potential. **C** Differential expression of immune checkpoint genes between high- and low-risk groups. **D** Comparative analysis of drug sensitivity (assessed by IC50 values) between different risk groups

In the immune function analysis, the high-risk group showed significantly elevated scores for most immune functions, except for Type I and Type II interferon responses, indicating that this group may be in a heightened state of immune activation or inflammation. In contrast, the low-risk group had higher scores for Type I and Type II interferon responses, suggesting a more effective anti-tumor immune response. These findings highlight distinct differences in immune functional profiles between the two risk groups (Fig. 10A).

TIDE score analysis revealed that the high-risk group had significantly higher TIDE scores compared to the low-risk group (Fig. 10B), implying a greater potential for immune evasion in the high-risk group.

Immune checkpoint gene expression analysis identified 39 genes as significantly differentially expressed between the high- and low-risk groups. Most of these genes were upregulated in the high-risk group, with the exception of IDO2, which was elevated in the low-risk group (Fig. 10C).

Finally, drug sensitivity analysis, comparing IC50 values between the high- and low-risk groups for various drugs, showed significant differences for 83 drugs (Fig. 10D). The high-risk group exhibited greater sensitivity to most drugs, suggesting that these patients may benefit more from specific targeted therapies or chemotherapeutic agents.

### Validation of hypoxia-related genes

**Fig. 11:**
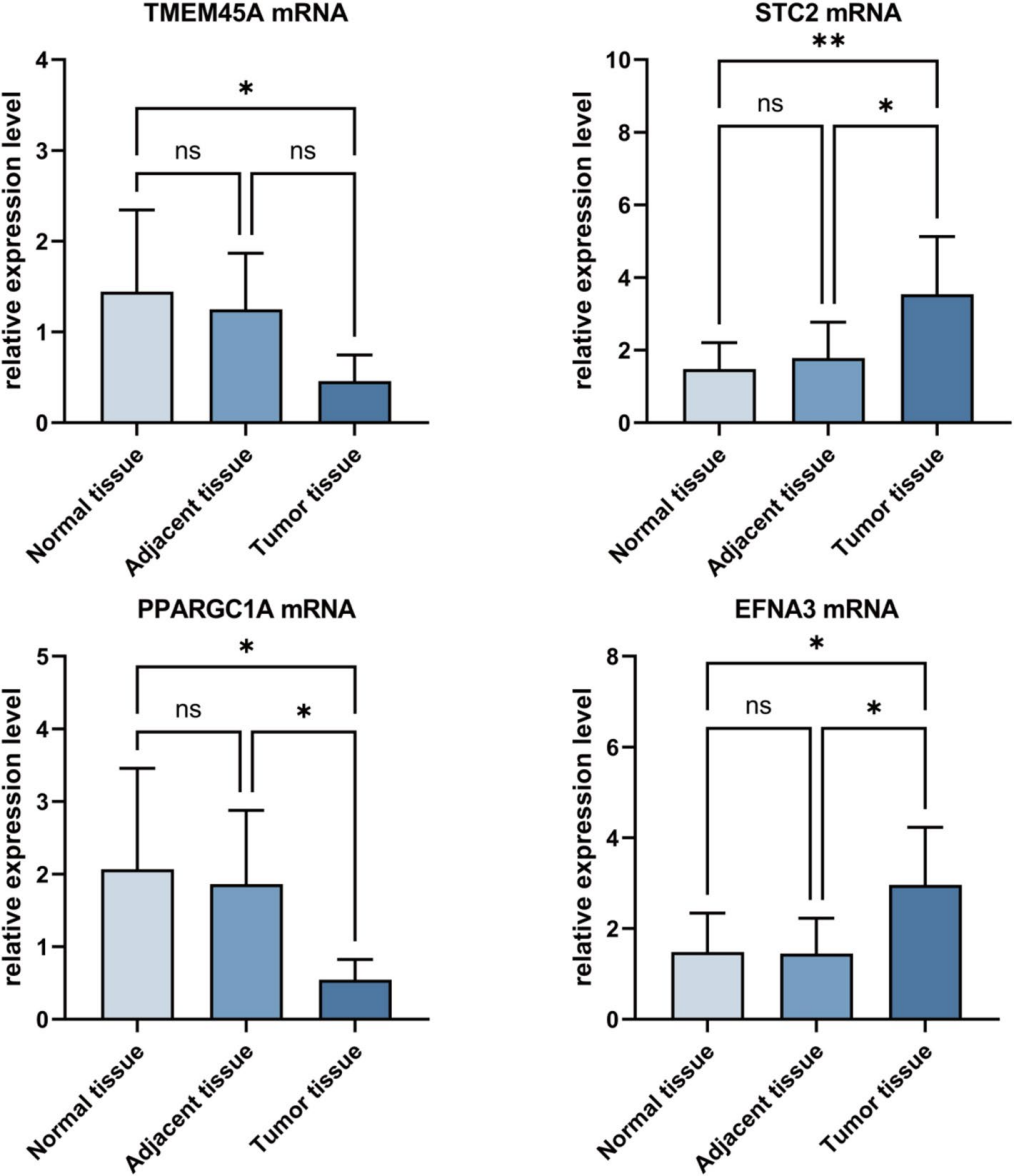
qRT-PCR analysis of selected gene expression in human liver tissues

To further validate the expression patterns of the four hypoxia-related genes (TMEM45A, PPARGC1A, EFNA3, and STC2) in human HCC tissues, qRT-PCR were conducted using a self-collected cohort of human tissue samples. Tumor and paired adjacent normal liver tissues collected during surgery underwent RNA-seq analysis, and a volcano plot was generated (Fig. 11A). The analysis revealed that all four genes exhibited differential expression patterns consistent with the TCGA-LIHC cohort: EFNA3 and STC2 were significantly upregulated in tumor tissues, while TMEM45A and PPARGC1A were downregulated, further supporting their role as hypoxia-related prognostic genes.

To strengthen the validation, qRT-PCR analysis was performed on normal liver tissues, adjacent non-tumorous tissues, and HCC tissues (Fig. 11B). The results showed that EFNA3 and STC2 had the highest expression levels in tumor tissues, followed by adjacent tissues, with the lowest levels in normal liver tissues. Conversely, TMEM45A and PPARGC1A exhibited an inverse pattern, showing highest expression in normal liver tissues, intermediate levels in adjacent tissues, and significantly lower expression in tumor tissues. These expression patterns aligned with both model predictions and public database analyses, indicating stable and biologically significant differential expression across various stages of hepatocarcinogenesis.

In conclusion, qRT-PCR validation confirmed the altered expression patterns of TMEM45A, PPARGC1A, EFNA3, and STC2 in HCC tissues. These findings reinforce the clinical relevance and robustness of the hypoxia-related gene model and provide a strong foundation for future mechanistic studies.

## Discussion

In this study, we developed and validated a novel hypoxia-related gene–based prognostic model for hepatocellular carcinoma (HCC) using the TCGA cohort and two independent validation cohorts, GSE14520 and GSE116174. The model comprises four key genes—TMEM45A, PPARGC1A, EFNA3, and STC2. Our results demonstrated that patients in the high-risk group had significantly poorer overall survival (OS) than those in the low-risk group. Moreover, the model exhibited robust performance and good generalizability across different cohorts, as evidenced by consistent C-index values, AUCs, and Kaplan–Meier survival analyses. These findings are highly consistent with accumulating evidence highlighting the critical role of hypoxia in HCC progression, prognosis, and remodeling of the tumor immune microenvironment.

Hypoxia is a critical pathological feature of HCC and promotes tumor angiogenesis, epithelial–mesenchymal transition (EMT), tumor cell invasion, and therapeutic resistance through the upregulation of transcription factors such as HIF-1α, ultimately leading to poor patient prognosis [18]. Patients in the high-risk group identified in our study may reflect tumors under more severe hypoxic stress, thereby exhibiting unfavorable characteristics in metabolism, proliferation, and immune evasion.

Immune microenvironment analysis revealed significantly increased M0 macrophage infiltration and elevated expression of multiple immune checkpoint genes in the high-risk group. Increased expression of PDCD1 (PD-1), CD274 (PD-L1), CTLA4, LAG3, TIGIT, and HAVCR2 (TIM-3) suggests that the high-risk group may represent an immunologically distinct HCC subtype. Such tumors may theoretically derive greater benefit from immune checkpoint blockade therapies. However, elevated checkpoint expression alone does not necessarily predict treatment response, as similar transcriptional patterns may also reflect immune exhaustion, adaptive immune resistance, or dysfunctional T-cell infiltration. Therefore, although our findings suggest that the hypoxia-related risk signature may help identify immunologically distinct HCC subgroups, its utility for predicting responses to immunotherapy or combination treatment strategies requires further validation in cohorts with detailed treatment and response information. Furthermore, because these findings were primarily derived from transcriptome-based computational inference, they cannot reliably distinguish productive antitumor immunity from functionally suppressed immune states. Previous studies have emphasized that computational immune deconvolution may not fully capture the spatial organization, functional states, and protein-level expression patterns of immune cells within the tumor microenvironment. Therefore, validation using multiplex immunohistochemistry, spatial transcriptomics, or single-cell sequencing is warranted to further clarify the immune landscape identified by our model [19–21].

Our analyses indicate that the hypoxia-related risk score is an independent predictor of overall survival, even when accounting for established clinical variables such as TNM stage, tumor size, and AFP levels. Subgroup analyses further revealed that the risk score stratifies patients within the same tumor stage, underscoring its ability to capture additional prognostic information beyond conventional staging. These findings suggest that the risk score reflects underlying hypoxia-driven biology rather than merely indicating advanced-stage disease. Future prospective studies integrating clinical, molecular, and functional hypoxia readouts will be necessary to fully validate its utility as a complementary biomarker for HCC prognosis.

Compared with previously reported hypoxia-related prognostic models for HCC, the model constructed in this study is structurally concise, consisting of only TMEM45A, PPARGC1A, EFNA3, and STC2, yet it demonstrated stable predictive performance and high clinical feasibility across the training cohort and two independent validation cohorts. Although some previously published multigene models achieved favorable AUC values, their relatively high complexity may limit practical clinical application. In contrast, our model achieves a favorable balance between simplicity, feasibility, and robustness, highlighting its potential value for clinical translation.

Notably, all four genes included in the prognostic signature are functionally linked to hypoxia-associated biological processes and collectively represent complementary dimensions of cellular adaptation to oxygen deprivation. TMEM45A has been identified as a HIF-1α–regulated hypoxia-responsive gene and contributes to hypoxia-induced chemoresistance in hepatoma cells [22]. EFNA3 has been reported as a hypoxia-responsive gene regulated through HIF-dependent pathways and participates in angiogenesis and metastatic progression [23]. STC2 is a classical hypoxia-inducible target gene involved in cellular adaptation to oxygen deprivation, endoplasmic-reticulum stress tolerance, and tumor aggressiveness [24]. In contrast, PPARGC1A serves as a master regulator of mitochondrial biogenesis and oxidative metabolism. Reduced PPARGC1A expression may indicate mitochondrial dysfunction and a shift toward glycolytic metabolism, a hallmark of hypoxia-associated metabolic reprogramming in cancer cells [25]. Although no definitive regulatory axis linking these four genes has been established, they converge on interconnected processes including hypoxia adaptation, metabolic remodeling, angiogenesis, cellular stress responses, and tumor survival. Consistent with this interpretation, PPI-network and enrichment analyses highlighted pathways associated with hypoxia-related metabolism and cellular adaptation, providing biological support for the prognostic relevance of the four-gene signature.

Beyond their association with hypoxia, accumulating evidence supports important roles of PPARGC1A and STC2 in HCC progression. PPARGC1A has been reported to regulate mitochondrial oxidative phosphorylation, fatty acid metabolism, and cellular energy homeostasis in HCC [26]. Reduced PPARGC1A expression is associated with impaired mitochondrial function and unfavorable prognosis in liver cancer [27, 28]. In contrast, STC2 has been shown to promote HCC cell proliferation, migration, invasion, and resistance to cellular stress through activation of multiple oncogenic signaling pathways [29]. Elevated STC2 expression has also been associated with advanced tumor stage and poor clinical outcome in patients with HCC [30]. These findings further support the biological relevance of PPARGC1A and STC2 in the prognostic signature identified in the present study.

Mechanistic analyses indicated that the differentially expressed genes in the high-risk group were significantly enriched in pathways related to metabolic reprogramming, including xenobiotic metabolism, steroid hormone biosynthesis, and the PPAR signaling pathway, as well as pathways associated with cell-cycle regulation, EMT, and MYC target genes. Together with the immune-related findings, these results suggest that the high-risk group may represent a distinct hypoxia-driven HCC subtype characterized by enhanced metabolic activity, increased invasiveness, and pronounced immune evasion. However, these findings should be interpreted as hypothesis-generating rather than definitive mechanistic evidence. Although our findings support a potential link between hypoxia-related gene expression, immune remodeling, and unfavorable prognosis in HCC, the underlying causal relationships remain speculative. Future studies incorporating gene knockdown or overexpression under hypoxic conditions, immune-cell co-culture systems, organoid models, and in vivo validation are required to establish causality and further elucidate the molecular mechanisms through which hypoxia contributes to immune remodeling and tumor progression [17].

From a clinical perspective, the hypoxia-related risk score demonstrated independent prognostic value beyond routine clinical variables. However, although calibration analysis was performed for the nomogram, clinically actionable risk thresholds and decision-curve analyses could not be fully established because detailed treatment information was unavailable in the public cohorts. Therefore, the present model should currently be regarded as a prognostic stratification tool rather than a treatment-decision instrument, and prospective studies are required to determine how specific risk categories can be incorporated into clinical workflows.

Several limitations of this study should be acknowledged. First, although qRT-PCR validation confirmed the differential expression of TMEM45A, PPARGC1A, EFNA3, and STC2 in HCC tissues, the validation cohort was relatively small (12 paired samples) and limited to mRNA expression analysis. Protein-level validation, including immunohistochemical assessment of protein expression and tissue localization, was not available. Furthermore, stratified analyses according to clinicopathological characteristics such as tumor stage, vascular invasion, and recurrence status could not be reliably performed because of the limited sample size. Future studies involving larger and clinically well-annotated cohorts are warranted to further investigate the clinicopathological significance of these genes.

Second, due to incomplete treatment and follow-up information in public cohorts, the predictive value of this model for treatment response and recurrence-related outcomes, including disease-free survival (DFS) and recurrence-free survival (RFS), could not be comprehensively evaluated. Third, the immune infiltration and drug-sensitivity analyses in this study were primarily based on transcriptomic computational inference. Although significant differences in predicted immune landscapes and IC50 values were observed between risk groups, these findings do not constitute direct experimental evidence and should therefore be interpreted with caution. Validation using immunohistochemistry, flow cytometry, spatial transcriptomics, cell-based drug-response assays, or patient-derived models will be necessary to confirm these observations [31, 32].

Fourth, although the prognostic signature was validated in multiple independent public cohorts, the tissue validation cohort was derived from a single center and included a limited number of patients. Given the recognized heterogeneity of HCC across different etiological backgrounds, including hepatitis B virus infection, hepatitis C virus infection, alcohol-related liver disease, and metabolic dysfunction-associated steatotic liver disease, the generalizability of the present model requires further evaluation. Future multicenter studies involving larger and geographically diverse populations are necessary to determine the robustness and clinical applicability of this signature[33]. Moreover, the four-gene signature was generated using predefined differential-expression thresholds and feature-selection procedures. Although these methods are widely accepted, alternative thresholds or machine-learning strategies may produce partially different gene sets. Therefore, additional validation using independent datasets and alternative analytical approaches is warranted to further confirm the robustness of the present model [34, 35].

Importantly, although the present model demonstrates robust prognostic performance across multiple retrospective cohorts, its clinical utility should currently be interpreted as exploratory rather than practice-changing. Prospective multicenter clinical validation is required before integration into routine clinical decision-making.

Overall, the hypoxia-related prognostic model developed in this study effectively stratifies patients with HCC into high- and low-risk groups and demonstrates robust stability and predictive performance across multiple independent cohorts. The high-risk group may represent a distinct hypoxia-driven HCC subtype characterized by metabolic reprogramming, enhanced invasiveness, and immune evasion. This model shows promising potential for clinical prognostic assessment and risk stratification and may provide a useful framework for future studies exploring personalized therapeutic strategies in HCC.

## Conclusion

We established and validated a novel four-gene hypoxia-related prognostic signature consisting of TMEM45A, PPARGC1A, EFNA3, and STC2 for hepatocellular carcinoma. The model demonstrated stable prognostic performance across multiple independent cohorts and served as an independent predictor of overall survival. High-risk patients exhibited distinct biological characteristics associated with hypoxia, including metabolic reprogramming, increased invasiveness, immune microenvironment remodeling, and elevated immune checkpoint expression. These findings suggest that the proposed signature may provide valuable information for prognostic assessment and risk stratification in HCC. Nevertheless, additional mechanistic studies and prospective clinical validation are required before routine clinical application. Our study provides new insights into the relationship between hypoxia and HCC progression and offers a promising framework for future precision oncology research.

## Supplementary Information

Supplementary Material 1.

Supplementary Material 2.

Supplementary Material 3.

Supplementary Material 4.

Supplementary Material 5.

## Data Availability

The datasets analyzed in this study were obtained from publicly available repositories. Specifically, data were sourced from The Cancer Genome Atlas (TCGA, https://www.cancer.gov/tcga), Gene Expression Omnibus (GEO, https://www.ncbi.nlm.nih.gov/geo/), dbEMT database (for EMT-related gene lists, https://dbemt.bioinfo-minzhao.org/), STRING database (for protein–protein interaction data, https://string-db.org/), MSigDB (for functional enrichment analysis gene sets,https://www.gsea-msigdb.org/gsea/msigdb), and TIDE (for immune response prediction, https://tide.dfci.harvard.edu/). The specific datasets utilized include TCGA-LIHC and GEO accession numbers GSE14520 (https://www.ncbi.nlm.nih.gov/geo/query/acc.cgi?acc=GSE14520) and GSE116174 (https://www.ncbi.nlm.nih.gov/geo/query/acc.cgi?acc=GSE116174). All processed data are included in the article and its supplementary material. Further inquiries can be directed to the corresponding author(s).

## Declarations

### Accordance statement

All methods were performed in accordance with relevant institutional, national, and international guidelines and regulations.
