# Supplementary material for: A novel hypoxia-related signature for predicting prognosis, immune characteristics, and therapeutic response in hepatocellular carcinoma
Source: Discov Oncol. 2026 Aug 7;17:1168. doi: 10.1007/s12672-026-05691-w (PMC13469028; doi:10.1007/s12672-026-05691-w)
Supplement: Supplementary file 1 [file 12672_2026_5691_MOESM4_ESM.docx]

**Figure S1** The AUC values of each model are 1 year, 2 years, and 3 years.

AUC (Area Under Curve), HR (Hazard Ratio).

Figure S2 The AUC values of our prognostic model compared with other hypoxia-related prognostic models at 1 year, 3 years, and 5 years in each dataset.AUC（Area Under Curve）
