# Supplementary material for: A novel hypoxia-related signature for predicting prognosis, immune characteristics, and therapeutic response in hepatocellular carcinoma
Source: Discov Oncol. 2026 Aug 7;17:1168. doi: 10.1007/s12672-026-05691-w (PMC13469028; doi:10.1007/s12672-026-05691-w)
Supplement: Supplementary file 2 [file 12672_2026_5691_MOESM5_ESM.docx]

**Supplementary Figure S1. Flowchart of the construction of the hypoxia-related prognostic signature in HCC.**

**Supplementary Figure S2. Venn diagram identifying differentially expressed hypoxia-related genes in the TCGA-HCC cohort.**

| **Supplementary Table S1. Differentially expressed hypoxia-related genes identified in the TCGA-HCC cohort (n = 65).** | | | | | | | |
| --- | --- | --- | --- | --- | --- | --- | --- |
| **SYMBOL** | | | | | | | |
| MT1E | | | GRHPR | | | BGN | |
| DCN | | | ANGPTL4 | | | F3 | |
| PLAC8 | | | PNRC1 | | | CP | |
| SRPX | | | TPBG | | | EDN2 | |
| FOS | | | SLC2A3 | | | HMOX1 | |
| MT2A | | | ALDOA | | | PLIN2 | |
| PCK1 | | | LOX | | | NFIL3 | |
| FBP1 | | | STC1 | | | ERRFI1 | |
| TMEM45A | | | P4HA2 | | | AKAP12 | |
| ENO3 | | | CAVIN3 | | | JUN | |
| CCN5 | | | ANXA2 | | | HAS1 | |
| GCK | | | PPFIA4 | | | ADM | |
| SERPINE1 | | | DTNA | | | MAFF | |
| IGFBP3 | | | INHA | | | CITED2 | |
| CCN1 | | | HOXB9 | | | BHLHE40 | |
| NOCT | | | KIF5A | | | PFKFB3 | |
| ZFP36 | | | EFNA3 | | | KLF6 | |
| ALDOB | | | SLC2A5 | | | RORA | |
| IL6 | | | CA12 | | | PGM1 | |
| PPARGC1A | | | STC2 | | |  | |
| IGFBP1 | | | B4GALNT2 | | |  | |
| ATF3 | | | BCAN | | |  | |
| DUSP1 | | | GPC3 | | |  | |
| **Supplementary Table S2. Univariate Cox regression results of prognostic hypoxia-related genes** | | | | | | | |
| **No.** | **Gene** | **HR** | | **z** | **P value** | | **Lower 95% CI** |
| 1 | STC2 | 1.36 | | 4.78 | <0.001 | | 1.20 |
| 2 | EFNA3 | 1.39 | | 4.10 | <0.001 | | 1.19 |
| 3 | PPFIA4 | 1.76 | | 3.91 | <0.001 | | 1.33 |
| 4 | ANXA2 | 1.34 | | 3.70 | <0.001 | | 1.15 |
| 5 | PPARGC1A | 0.80 | | -3.68 | <0.001 | | 0.71 |
| 6 | LOX | 1.25 | | 3.21 | 0.001 | | 1.09 |
| 7 | ADM | 1.25 | | 3.21 | 0.001 | | 1.09 |
| 8 | HMOX1 | 1.23 | | 3.13 | 0.002 | | 1.08 |
| 9 | TMEM45A | 1.16 | | 3.00 | 0.003 | | 1.05 |
| 10 | PFKFB3 | 1.17 | | 2.93 | 0.003 | | 1.05 |
| 11 | IGFBP3 | 1.19 | | 2.89 | 0.004 | | 1.06 |
| 12 | ALDOB | 0.92 | | -2.60 | 0.009 | | 0.86 |
| 13 | MAFF | 1.23 | | 2.57 | 0.010 | | 1.05 |
| 14 | SERPINE1 | 1.13 | | 2.56 | 0.010 | | 1.03 |
| 15 | SLC2A5 | 1.15 | | 2.54 | 0.011 | | 1.03 |
| 16 | FBP1 | 0.90 | | -2.42 | 0.015 | | 0.82 |
| 17 | ALDOA | 1.29 | | 2.33 | 0.020 | | 1.04 |
| 18 | PCK1 | 0.93 | | -2.30 | 0.022 | | 0.87 |
| 19 | GRHPR | 0.80 | | -2.25 | 0.025 | | 0.67 |
| 20 | STC1 | 1.17 | | 2.20 | 0.028 | | 1.02 |
| 21 | RORA | 0.82 | | -2.02 | 0.044 | | 0.68 |

**Supplementary Figure S3. LASSO regression coefficient profiles of 21 prognostic hypoxia-related genes.**

**Supplementary Figure S4. Cross-validation for tuning parameter selection in the LASSO Cox regression model.**

| **Supplementary Table S3. Genes retained by LASSO Cox regression and corresponding coefficients** | | |
| --- | --- | --- |
| **Gene** | **LASSO coefficient** | **Final status** |
| STC2 | 0.187 | Retained |
| EFNA3 | 0.178 | Retained |
| PPARGC1A | -0.157 | Retained |
| ADM | 0.006 | Removed |
| HMOX1 | 0.023 | Removed |
| TMEM45A | 0.058 | Retained |
| MAFF | 0.027 | Removed |
| SLC2A5 | 0.047 | Removed |
| STC1 | 0.044 | Removed |

| **Supplementary Table S4. Multivariate Cox regression results of the final four-gene prognostic model.** | | | | |
| --- | --- | --- | --- | --- |
| **Gene** | **Coefficient** | **HR** | **95% CI** | **P value** |
| TMEM45A | 0.102 | 1.11 | 1.00–1.22 | 0.046 |
| PPARGC1A | -0.202 | 0.82 | 0.72–0.92 | 0.001 |
| EFNA3 | 0.214 | 1.24 | 1.05–1.46 | 0.012 |
| STC2 | 0.262 | 1.30 | 1.14–1.48 | <0.001 |
